# 

**Published:** 1855-10

**Authors:** 


### CONTENTS OF VOLUME IX.

Amalgam for Filling Teeth........ 308
American Society of Dental Surgeons, and American Dental Convention,........ 77
Baneful Effects of Saleratus and Cream of Tartar,........ 322
Caries of the Teeth. By J. TAFT, D. D. S.,........ 34
Case of Hypertrophy of the Gums, in the Practice of Professor GROSS. Reported by WM H. GODDARD, D. D. S., Louisville, Ky........ 276
Cases of Alveolar Abscess........ 175
Causes of Caries.—Continued from October Number........ 392
Chlorine. (CL—35.42.) By G. WATT, D. D. S., M. D., Xenia, Ohio,........ 50
Connection of Dentition in Children with Nursing. Clinical Lecture at the Hotel Dieu. By M. TROUSSEAU........ 415
Correspondence. N. B. SLAYTON........ 129
Correspondence,........ 206
Correspondence,........ 327
Correspondence,........ 397
Dental Abstracts,........ 192
Dental Education. By H. R. SMITH, M. D., D. D. S........ 341
Dr. J. M. LOCKE's Report on his Analysis of Delabarre's, Drs. Allen and Hunter's Formulas for Continuous Gum Work,........ 59
Drying Cavities Preparatory to Filling. TAFT & WATT........ 72
Editorial........ 110
Editorial,........ 213
Editorial,........ 330
Editorial,........ 000
Executive Clemency—Pardon of Dr. Beale........ 171
Extracts from the Records of the Boston Society for Medical Observation. By S. P. SPRAGUE, M. D., Secretary........ 186
Foreign Body in the Œsophagus,........ 419
From an Introductory Lecture on Dental Chemistry. By GEO. WATT........ 346
Gutta Percha. SLAYTON........ 73
Gutta Percha Gums,........ 358
Highly Interesting to Dentists,........ 106
Humbugiana. By ONE WHO HAS BEEN "SOLD."........ 122
Inflammation of the Dentine. By J. HAMILL........ 252
Lectures on the Duties and Proprieties of the Dental Practitioner. By Professor E. TOWNSEND, D. D. S., Philadelphia,........ 1
Local Anæthesia by Congelation in Dental Surgery. By RICHARD QUINTON, London.... 133
Local Anæsthesia,........ 299
Local Anæsthesia by Congelation. By H. L. BURPEE, Williamsburgh, L. I........ 408
Management of Light in the Performance of Dental Operations. By J. M'QUILLEN, D. D. S.,........ 106
Minutes of the Fourth Annual Meeting of the Ohio Dental College Association........ 229
Miscellaneous Items,........ 209
Miscellaneous Items,........ 326
Miscellaneous Items,........ 438
Neuralgia Facial, or Tic Douloureux. By T. D. THOMPSON, D. D. S., Providence, R. I... 189
On the Cure of Toothache, and a Method of Treating Exposed Nerves. By DONALDSON MACKENZIE, ESQ., Surgeon-Dentist, London........ 179
On the Metal Aluminum,........ 295
On the Use of Amalgam for Filling Teeth. By ELISHA TOWNSEND........ 156

Operation for the Removal of a Large Artificial Tooth-Plate from the Pharynx.—Recovery. Case under the Care of Mr. Cock........ 410
Pivoting Teeth. By John Coghlan, M. D., M. R. C. S. Eng. &c........ 305
Preparation of the Mouth for Artificial Teeth. By H. R. Smith, M. D., D. D. S.,........ 389
Quackery. By J. W. Salisbury, M. D........ 315
Report of a Case of Nervous Disease. By Harry Dove, M. R. C. S., &c.,........ 162
Report of Discussions of the Mississippi Valley Associatton,........ 259
Removal of a Tumor from the Hyoid Bone, by Dr. A. Dunlap. Reported by Dr. C. N. Woodward........ 391
Sketches of the Knowledge of Teeth. The Origin of Medical Science. By George Hayes, Esq.. Dentist, Conduit Street, Hanover Square, London,........ 398
Specimens of Dental Pathology, Gutta Percha, &c. By Alexander M. Holmes, D. D. S., Morrisville, Madison County, New York........ 405
Teeth of Nations. Introduction........ 354
The Dental Student's Preparatory Education.—An Address. By Rev. B. P. Aydelott, D. D.,........ 221
The Dental Obturator. By A. S. Talbert, D. D. S., Lexington, Ky., ........ 70
The Paris Exhibition,........ 300
The Use of Amalgams for Filling Teeth. An Inaugural Dissertation submitted to the Trustees and Faculty of the Ohio College of Dental Surgery, for the Degree of Doctor of Dental Surgery. By E. Osmond........ 364
Three Cases from My Note Book. By S. H. Harvey, M. D., Washington, Ark........ 190
Treatment of Alveolar Abscess. By Professor White........ 104
Treatment of Dental Caries when Complicated with an Irritable or Exposed Pulp. By James Taylor,........ 42
Treatment of Dental Caries, &c.,—Continued,........ 113
Treatment of Dental Caries, &c.—Concluded........ 371
Twelfth Annual Meeting of the Mississippi Valley Association of Dental Surgeons,........ 236
Valedictory Address to the Graduates of the Ohio College of Dental Surgery, of the Session of 1855–6. By Professor J. Taft........ 282
Western Dental Society,........ 395

### RECEIPTS FOR THE REGISTER.

| | | | |
|---|---|---|---|
| Dr. Garner,.. | $3 00 | Dr. D. L. Pratt, | 5 00 |
| Dr. N. M. Hicks, | 4 25 | Mr. J. D. Chevalier, | 25 00 |
| Dr. C. R. Thomas, | 4 00 | Dr. J. B. Williams, | 2 25 |
| Dr. J. Murry, | 5 00 | Dr. J. Pattan, | 4 00 |
| Dr. D, Dougherty, | 2 00 | Dr. A. L. Brown, | 12 50 |
| Dr H. Munro, | 2 00 | Dr. J. Hough, | 7 00 |
| Dr. —. Poore | 2 00 | Dr. R. T. Willard, | 5 00 |
| Dr. James Gillespie, | 2 50 | Messrs. Abby & Son, | 18 00 |
| Dr. A. S. Talbert, | 2 50 | Dr. A. Clark, | 3 00 |
| Dr. C. C. Thimme, | 3 00 | | |

CONTENTS—ORIGINAL COMMUNICATIONS.

American Society of Dental Surgeons, and American Dental Convention ..... 77

Selected Articles ..... 104

Editorial ..... 110

### PROSPECTUS.

### THE DENTAL REGISTER OF THE WEST

Has now closed its Eighth yearly salutation to the Profession. It was started for a specific object, the diffusion of scientific and practical knowledge.

Those who have regularly read its pages can see how this object has been accomplished. We can only say, that on a retrospect, we feel that our labor has not been in vain ; that while we cannot claim all that we desired, yet we see enough to give us cheerful anticipations for the future.

The past eight years have been prolific in Dental improvement and progress, and we feel that our profession now occupies a position far more favorable for advancement.

When the Register was started, eight years since, we had no Western periodical belonging to the Profession and it was with much doubt and hesitation that it would be sustained. It has, however, passed its infancy, and from a small forty-eight page octavo quarterly, it has grown to almost double that size. The wants of the Profession and the amount of readable matter supplied for its pages now demand a still greater enlargement. To meet this want, and supply the profession with such a periodical as is needed in this great valley, we have consented to enlarge still further the Register and issue quarterly a work of one hundred and twelve octavo page at $3·00 per annum.

The Mississippi Valley Association of Dental Surgeons, at their last meeting requested this enlargement and also the increase of price.

The Register will be issued as heretofore, quarterly, on the first of October, January, April, and July. It will be devoted to the interests of Dental Science, and will be made up of original articles; also selected articles from Dental and Medical journals.

The department of Dental Abstracts will still be maintained—using this as a means of condensing that which is most valuable.

We shall as far as possible maintain a department on Dental Chemistry and Metallurgy, and design in each number to appropriate a few pages to a critical review of Dental and Medical publications. This will be particularly applied to all which relates to Dental Surgery. This review we mean shall be impartial, manly and courteous—handling false views and erroneous practice without favor—leaving the truths alone of science to exert their mighty influence in the continued elevation of our Profession.

We hope that eight years labor in connection with the Register, will justify us in saying that our aim is the advancement of Dental Science. If we ever felt that every energy and talent should be thus exerted, we feel it now, and hence, in buckling on our armor for the increased labor assumed, we say to the profession, "Excelsior, Excelsior," and hope that every Dental practitioner will send back the cheerful answer, "Onward, Onward; *we* come to the rescue."

In conclusion, we bespeak, from the profession regular contributions for its pages. Practical, appropriate and concise articles will be thankfully received.

All commuuications should be addressed "Dental Register, Cincinnati, Ohio," or "Dr. James Taylor." The Post Office regulations should be remembered.

JAS. TAYLOR.

### TO THE PROFESSION.

I take this method to announce to the profession that I have succeeded in giving to Gutta Percha, that permanent gum color, which has so long been wanted for dental purposes.

The Gutta Percha used is the pure gum heretofore only used for chemical purposes, and as prepared requires from 170° to 180° to bring it to a state ready for dental use. The color will stand the test of acids, alkalies, or any of the secretions of the mouth.

This article is offered to the profession especially for temporary operations, with the full confidence that it will last twice as long as such operations are designed to be worn.

For the manufacturing and coloring of this article, we have applied for letters patent, and have made an arrangement with Messrs. Jones, White, & McCurdy, Philadelphia, to manufacture and supply the profession with the same. Who will sell it to *only* those who present orders from myself or agents.

For the state of Ohio, Indiana, Kentucky, Illinois, Michigan, Wisconsin, Iowa, Missouri, Arkansas, Tennessee, Mississippi, and Louisiana, we have constituted Dr. Joseph Taylor of Cincinnati, sole agent, who will instruct those wishing to visit Cincinnati, for that purpose, in the mode of putting up the work, and fill up cirtificates, or orders for the purchase of the material; and also send out an agent or agents through those states for that purpose. The design being to let the profession have the full benefit of the improvement on very liberal terms.

N. B. SLAYTON.

Dr. M. W. S. Kendall is authorized by me to give instruction and fill orders for the purchase of the Gutta Percha in any of the states above named on such terms as may be agreed upon and will as soon as possible visit all the towns of importance in these states.

JOSEPH TAYLOR.

